# DNA aptamers that inhibit binding to human interleukin-17A and interleukin-20

**DOI:** 10.1039/d6ra00178e

**Published:** 2026-04-20

**Authors:** Ali Parvez, Kirsten Sully, Dana A. Baum

**Affiliations:** a Department of Chemistry, Saint Louis University 3501 Laclede Avenue St. Louis MO USA dana.baum@slu.edu

## Abstract

Cytokines interleukin-17 (IL-17) and interleukin-20 (IL-20) play important roles in inflammatory processes and autoimmune diseases. Using systematic evolution of ligands by exponential enrichment (SELEX), we have isolated DNA aptamers for each of these cytokines. For IL-17A, the best aptamers bound with *K*_d_ values as low as 4 nM, comparable to previously reported aptamers. IL-20 represents a novel target for aptamer identification and SELEX isolated aptamers with *K*_d_ values as low as 120 nM. Photo-crosslinking experiments confirmed aptamer binding to its cytokine target, most likely to the cytokine monomer. These novel DNA aptamers for IL-17 and IL-20 represent valuable tools for studying cytokine biology and developing potential therapeutics for inflammatory and autoimmune conditions like psoriasis and impaired wound healing.

## Abstract 2

DNA aptamers can inhibit binding to cytokines interleukin-17 (IL-17) and interleukin-20 (IL-20), which play important roles in inflammatory processes and autoimmune diseases.

## Graphical abstract

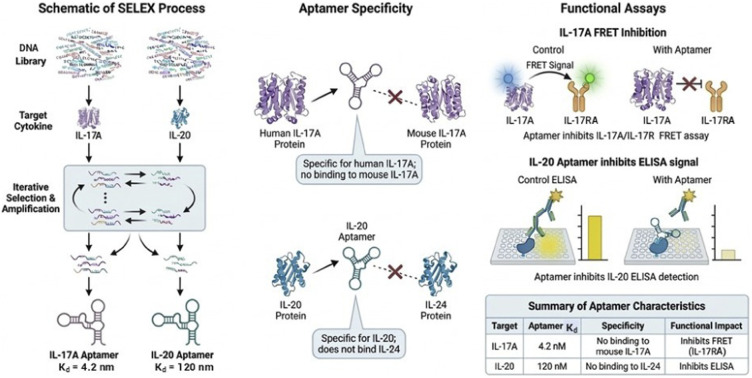

## Introduction

Cytokines have emerged as critical players in orchestrating immune and inflammatory responses, with specific families playing prominent roles in various pathological and physiological processes. Among these, interleukin 17 (IL-17) and interleukin 20 (IL-20) cytokines have garnered significant attention due to their distinct and pivotal contributions to immune regulation and disease pathogenesis. The IL-17 family, comprised of several structurally related cytokines, is primarily produced by a specialized subset of T-cells known as Th17 cells. These cytokines, including IL-17A, are essential for the host's defense mechanisms against bacterial and fungal infections, while their overproduction is linked to a host of autoimmune and inflammatory diseases, such as psoriasis, rheumatoid arthritis, and multiple sclerosis.^1,2^ IL-17's key function in recruiting neutrophils and stimulating the production of antimicrobial proteins underscores its importance in maintaining immune homeostasis.^3^ Similarly, IL-20, part of the IL-10 cytokine family, is involved in mediating inflammatory responses and promoting tissue repair and regeneration. The shared receptor usage within the IL-10 family enables IL-20 to exert its effects on immune regulation, tissue homeostasis, and host defense, although the precise mechanisms remain under active exploration.^4^ Research into these cytokines has propelled the development of targeted biological therapies aimed at modulating their activity to treat chronic inflammatory and autoimmune diseases.^5^ The overlapping yet distinct roles of IL-17 and IL-20 highlight the complexity of cytokine networks and their relevance in therapeutic interventions, heralding new avenues for treating conditions characterized by dysregulated cytokine activity.

**Fig. 1:**
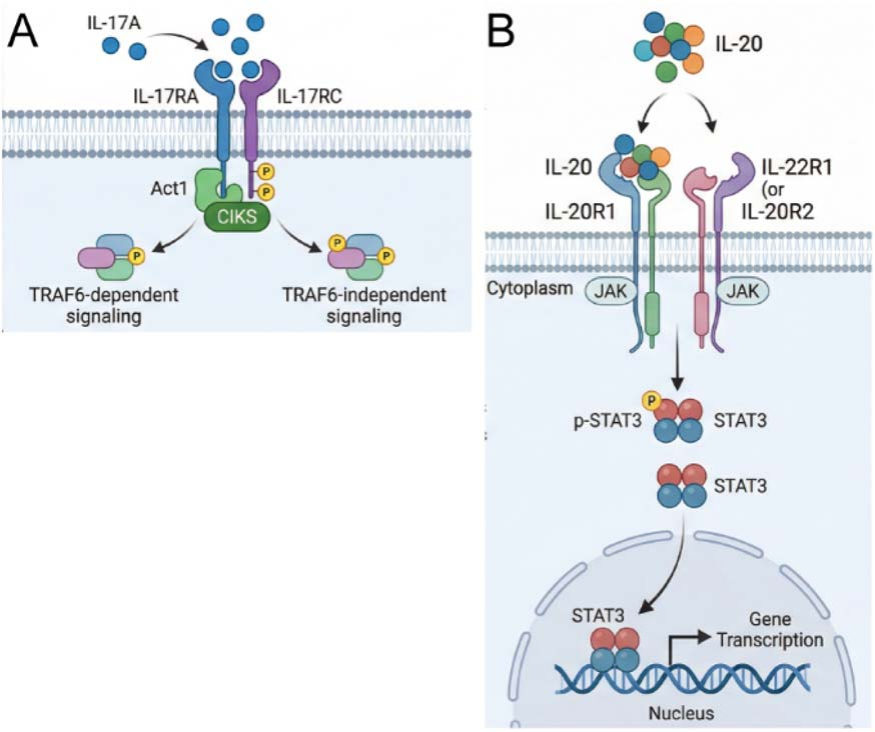
IL-17A and IL-20 signaling pathways. (A) IL-17A binds the IL-17RA/IL-17RC receptor complex, recruiting Act1/CIKS and triggering both TRAF6-dependent and TRAF6-independent downstream signaling cascades. (B) IL-20 engages a heterodimeric receptor (IL-20R1 with IL-22R1 or IL-20R2), activating JAK kinases that phosphorylate STAT3. Dimerized p-STAT3 translocates to the nucleus to drive target gene transcription.

IL-17 and IL-20 are homodimers that bind to a heterodimeric receptor complex for intracellular activation.^6,7^ These cytokines first bind to receptor complex a (Ra), which in turn recruits receptor complex b (Rb) for downstream activation of the JAK stat pathway (Fig. 1).^6,7^ Thus, approaches that can interfere with either receptor binding or dimer formation could serve as impactful therapeutic approaches for a range of disorders. For example, risankizumab (Skyrizi), an FDA-approved therapy for psoriasis,^8^ is a monoclonal antibody that targets interleukin 23 (IL-23), a downstream regulator of IL-17.^8^ Targeting both IL-17 and IL-20 could potentially provide a more comprehensive approach to modulating the inflammatory and proliferative responses in diabetic wound healing and psoriasis.

Rather than using antibodies, we seek to target IL-17A and IL-20 using aptamers. Aptamers are short, synthetic, single-stranded (ss) DNA or RNA sequences that display affinity and specificity for various targets, including chemical compounds, proteins, cells, and micro-organisms.^9^ Aptamers are identified through an iterative *in vitro* process known as the Systematic Evolution of Ligands by Exponential Enrichment (SELEX)^10^ or *in vitro* selection.^11^ While RNA^12–14^ and DNA^15^ aptamers have been identified for IL-17, to our knowledge, no aptamers have been reported for IL-20. Aptamers offer distinct advantages over antibodies, such as ease of synthesis and stability, and especially regarding DNA aptamers, cost.^16^ In this work, we sought to expand the aptamers available to bind to IL-17, while also identifying aptamers for IL-20 to provide new reagents for specifically recognizing and potentially inhibiting these two impactful cytokines.

## Results and discussion

**Fig. 2:**
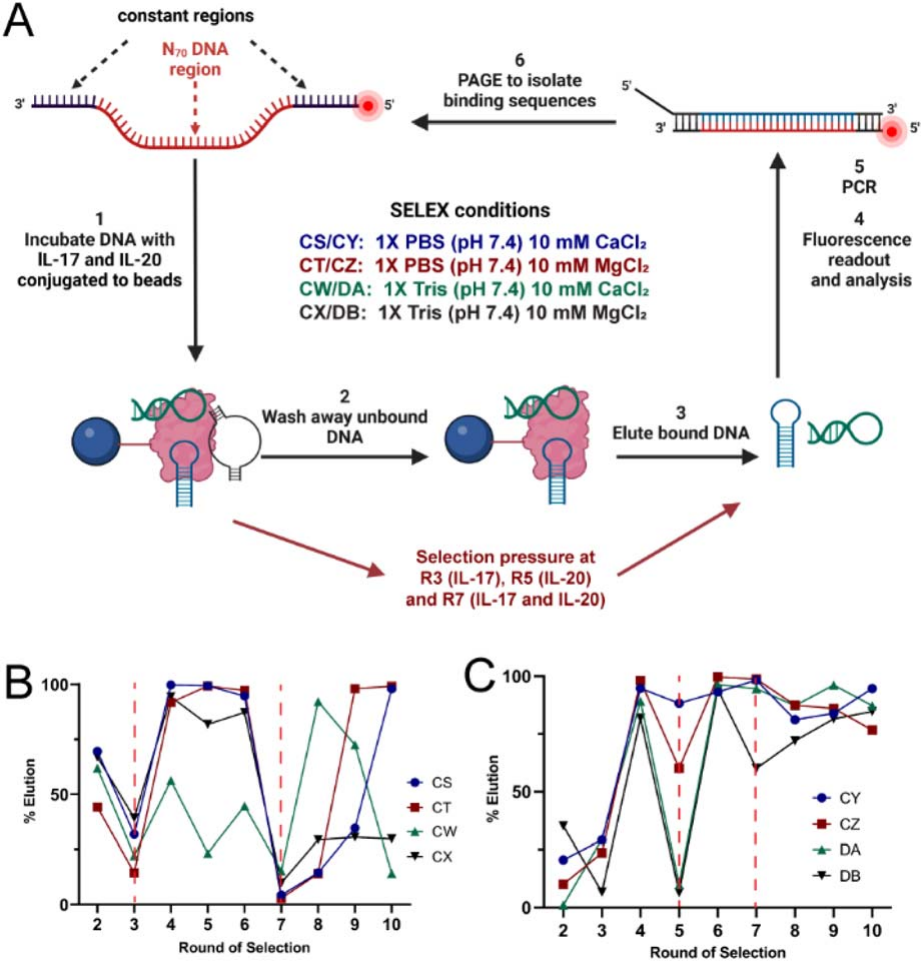
SELEX approach and progression for identifying aptamers for IL-17 and IL-20. (A) Aptamer identification begins with a DNA pool containing oligonucleotides with a region of 70 randomized nucleotides (designated N_70_) flanked by fixed primer binding regions, which is introduced to the immobilized cytokine target. Unbound DNA is washed away and bound DNA is then eluted using denaturing conditions. The recovered DNA is amplified by PCR and separated on denaturing PAGE to isolate the single-stranded binding sequences for the next round. Counter selections were performed as indicated to increase binding stringency for the desired target. (B) SELEX progress for IL-17-binding aptamers. Counter selection was performed at rounds 3 and 7. (C) SELEX progress for IL-20-binding aptamers. Counter selection was performed at rounds 5 and 7. Percent elution was calculated based on the fluorescence measured in the elutions compared to the total fluorescence in all washes and elutions.

SELEX for DNA aptamers for IL-17A and IL-20 was conducted for 10 rounds (Fig. 2). We used Tris and PBS as our selection buffers as these buffers are both commonly used in biological applications. Depending on downstream applications, one of these buffers may be preferred over the other and having aptamers that were selected in the desired buffer would be beneficial. For IL-17A selections (named CS, CT, CW, and CX to designate different selection buffer conditions, Table S1), counter selections were done at rounds three (R3) and seven (R7) to improve specificity for the desired target. The R3 counter selection was to remove sequences that were binding to the tether linking the target cytokine to the magnetic bead. The R7 counter selection challenged the sequences with IL-20 (our other cytokine target) and bovine serum albumin (BSA), which is used as a crowding agent to stabilize cytokines for biological applications. For those counter selection rounds, a significant drop in elution fluorescence was observed, indicating fewer binding sequences were recovered (Fig. 2B). Such a drop is not unexpected as sequences with less selective binding are eliminated and the elution fluorescence rebounded in the subsequent rounds. Selection CW consistently showed a lower elution fluorescence, which could be attributed to inconsistent binding of the few recovered oligonucleotides and ultimately failure to achieve an enriched collection of binding sequences. Selection CX showed enrichment until R7, but elution fluorescence failed to recover after the second counter selection. This observation could mean that the enriched binding sequences were capable of binding to IL-20 and/or BSA in additional to IL-17A and thus were removed in the R7 counter selection. Selections CS and CT, which both used PBS buffer at pH 7.4 but differed in the provided divalent metal ion, emerged as the more promising selections, with nearly 100% elution fluorescence at R10 (Fig. 2B).

For IL-20 selections (named CY, CZ, DA, and DB and differing in the selection buffer conditions, Table S1), counter selections were applied at rounds five (R5) and seven (R7). The R5 counter selection was to remove sequencing that were binding to the tether linking the target cytokine to the magnetic bead. The R7 counter selection challenged the sequences with IL-17A and BSA. Elution fluorescence decreases were observed for both counter selection rounds, but selection DA showed a significant drop only for R5, while selection DB showed significant drops for both R5 and R7 (Fig. 2C). However, the elution fluorescence rebounded for all selections by R9, indicating that binding sequences were enriched after these counter selections.

Eluted DNA oligonucleotides from R9 of selections CS and CT for IL-17A and R10 of selections CY, CZ, DA, and DB for IL-20 were cloned and sequenced (Tables S2–S4). The resulting sequences were compared and aligned to identify unique clones (Fig. S1 and S2). Unique clones were tested for binding in assays similar to what was done during the selection rounds (Tables S5 and S6). Clones that showed enhanced binding profiles were ordered as oligonucleotides (Table S7) for labelling and were tested in fluorescence polarization assays to determine binding affinity. Our isolated aptamers from selections CS and CT were compared to reported DNA aptamers for IL-17.^15^

**Fig. 3:**
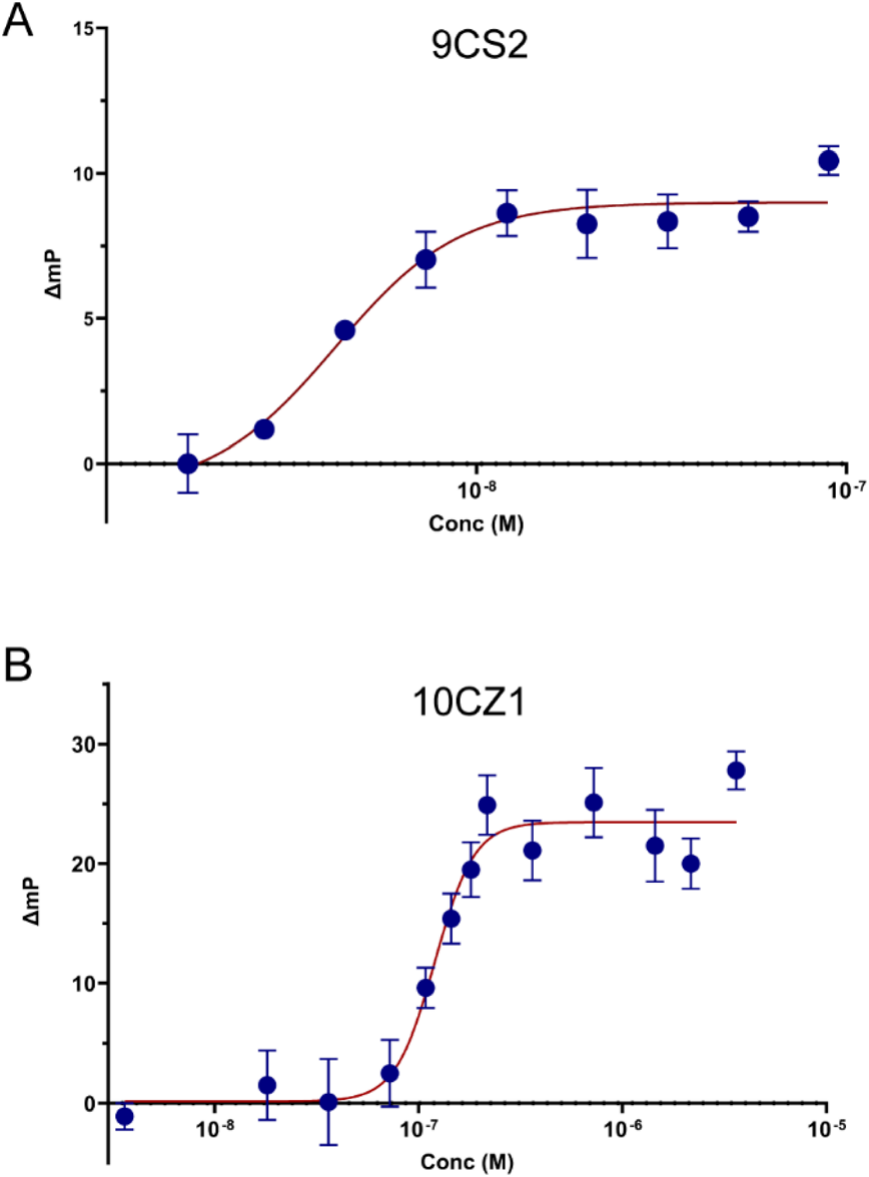
Determining aptamer binding constants using fluorescence polarization. Bodipy-TAMRA-labeled aptamer was incubated with increasing concentrations of cytokine target and the change in fluorescence polarization was measured. Aptamer 9CS2 binds IL-17 with a *K*_d_ of 4.2 nM (A). Aptamer 10CZ1 binds to IL-20 with a *K*_d_ of 120.2 nM (B). Error bars represent the standard deviation for *N* = 3 trials.

For IL-17A, aptamer 9CS2 emerged as the best binder with a *K*_d_ of 4.2 nM (Fig. 3A). Previously reported IL-17 aptamers^15^ were also tested with fluorescence polarization, resulting in a *K*_d_ of 3.1 nM for the full-length aptamer 2 and a *K*_d_ of 2.5 nM for the truncated version of aptamer 2 (Table S8). Thus our newly identified 9CS2 aptamer shows comparable binding to other reported IL-17 aptamers.^15^ Low nanomolar *K*_d_ values were found for six of our tested IL-17 aptamers, providing a collection of aptamers for future applications. Additionally, several of our isolated aptamers, the previously reported aptamer 1, and the truncated aptamer 1 produced binding curves that were not consistent with single binding events (Fig. S3). These aptamers were not studied further.

For IL-20, aptamer 10CZ1 emerged as the best binder with a *K*_d_ of 120.2 nM (Fig. 3B). Aptamers 10CY7 and 10DA18 were additional IL-20 aptamers identified under different buffer conditions and had similar *K*_d_ values of 304 nM and 174 nM (Fig. S4). Overall, our isolated IL-20 aptamers appear to have slightly weaker binding to their target, with binding constants that were in the low micromolar range, rather than the nanomolar values observed for our IL-17A aptamers. The same reaction conditions and approach were used for both of these targets, but differences in the targets maybe lead to different binding pockets and ultimately less effective aptamer binding for IL-20. However, these isolated sequences represent the first reported aptamers for IL-20.

**Fig. 4:**
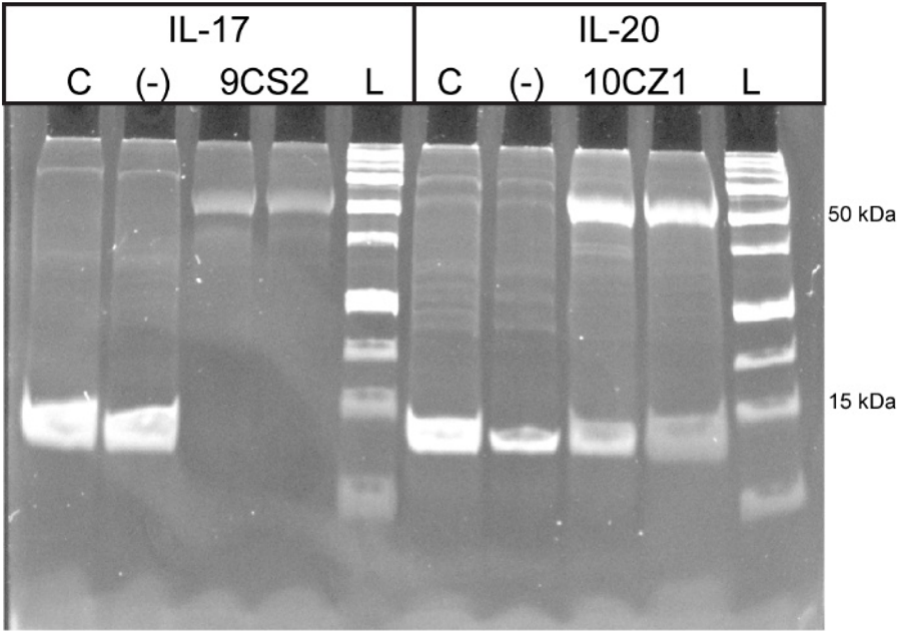
Demonstrating binding between isolated aptamers and target cytokines *via* photo-crosslinking. Migration of cytokines (C) was compared to cytokines incubated and photo-crosslinked with a non-binding sequence (−) or the isolated aptamer (9CS2 for IL-17 and 10CZ1 for IL-20). Samples and a protein ladder (L) were separated on 10% SDS-PAGE. Control cytokine lanes and non-crosslinked cytokines migrate just below 15 kDa, while cross-linked aptamer-cytokine complexes migrate near 50 kDa. Faint bands in the cytokine-only and negative control lanes are attributed to minor proteins that carried over during cytokine expression and purification.

To further characterize the binding between the aptamers and their target interleukins, we conducted photo-crosslinking assays with our two best aptamers, 9CS2 for IL-17 and 10CZ1 for IL-20 (Fig. 4). We first modified the aptamer with a photo-crosslinker. After allowing the aptamer/interleukin complex to form, we activated the crosslinker and separated the products on SDS-PAGE. As shown in Fig. 4, aptamer 9CS2 strongly crosslinked to IL-17. A non-binding control oligonucleotide failed to form a crosslinked complex, confirming that specific binding between the aptamer and IL-17 was necessary to form the crosslink. Aptamer 10CZ1 does not bind as strongly to its IL-20 target, as determined above in our binding assays, so while there is formation of a significant amount of aptamer/IL-20 crosslinked complex, there remains a small amount of unbound IL-20. These results confirm the binding interactions between the isolated aptamers and their target interleukins. Our protein expression and isolation, as well as the immobilization approach used for SELEX, likely presented the monomeric form of the interleukins for binding rather than the dimer that is responsible for signaling. Given the nature of the photo-crosslinker, we anticipate only observing crosslinking between the aptamer and the interleukin monomers.

While human IL-17A was our target, binding to the mouse version could be useful in different assays, including animal studies. Using fluorescence polarization, we assessed the binding of 9CS2, 9CS3, truncated aptamer 1, and truncated aptamer 2 with mouse IL-17A. All four of these aptamers showed, at best, weak binding to mouse IL-17 (Fig. S5), indicating our new IL-17 aptamers and the previously reported aptamers strongly prefer the human IL-17.

**Fig. 5:**
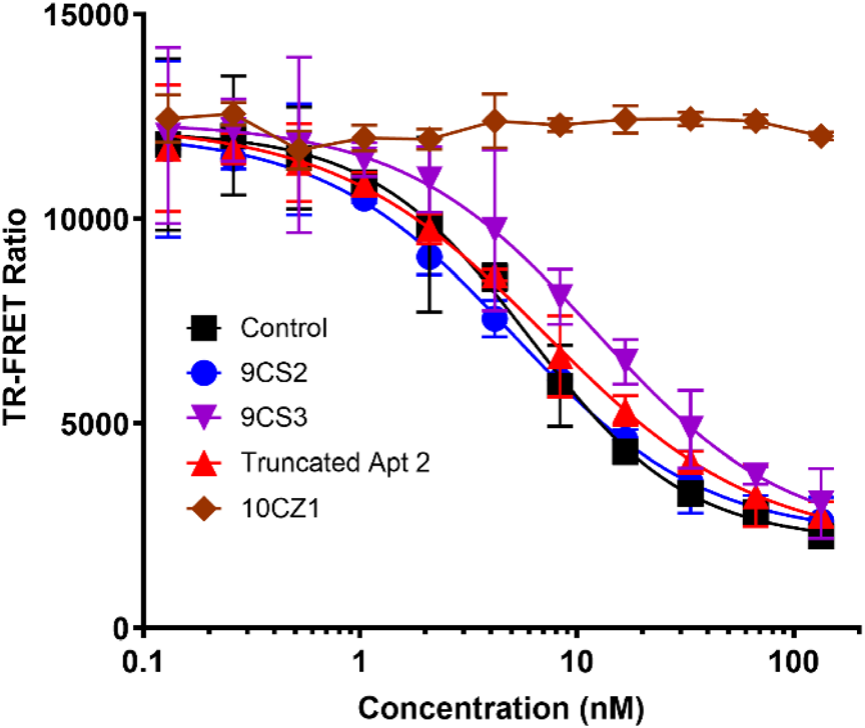
Demonstrating IL-17 aptamers prevent IL-17 binding to its receptor. Increasing concentrations of aptamers 9CS2, 9CS3, truncated Apt 2, and 10CZ1 were introduced to IL-17A labelled with the donor europium chelate and allowed to bind. Next the IL-17RA labelled with acceptor fluorophore was introduced and the fluorescence at 665 nm and 620 nm was recorded to calculate the TR-FRET ratio. The control was a neutralizing antibody provided with the kit (Acro Biosystems). Error bars represent the standard deviation for *N* = 3 trials.

As a final assessment of the IL-17 aptamers, we tested our aptamers as inhibitors that prevent IL-17A binding to its receptor IL-17RA. In this FRET-based assay, binding of an inhibitor prevents association of labelled IL-17A with labelled IL-17RA and reduces the FRET between the labels. If the aptamers do not bind to IL-17A or bind in a location that does not disrupt the interaction with the receptor, a high FRET signal is expected to be observed. Aptamers 9CS2 and 9CS3, as well as truncated aptamer 2, act as inhibitors to IL-17A binding to its receptor, with assay curves similar to those for the anti-IL17A antibody control (Fig. 5). In contrast, 10CZ1, which is specific for IL-20, does not produce any decrease in FRET, demonstrating it does not interfere with IL-17 binding to its receptor.

As IL-20 represents a new target for aptamers, we sought to better understand the structure of 10CZ1. Secondary structure prediction tools provide possible structures, but such programs do not account for any impact of target binding on the final aptamer structure. We performed DMS probing targeting Gs of the free aptamer and the bound aptamer to provide insights into the most likely regions of the aptamer to be involved in interleukin binding (Fig. S6). The results indicate changes in the aptamer structure upon IL-20 binding are predominantly within nucleotides 1–40, providing guidance for further work to minimize the aptamer structure.

Cytokine IL-24 shares 30% sequence homology with IL-20 and both IL-20 and IL-24 signal through the same two heterodimeric receptor complexes: IL-20R1/IL-20R2 and IL-22R1/IL-20R2, activating JAK/STAT pathways,^17–20^ so it was used to assess the specificity of the IL-20 aptamers in fluorescence polarization assays. Given this homology between IL-20 and IL-24, we anticipated there could be binding of our IL-20 aptamers to IL-24. Our best IL-20 aptamer, 10CZ1, showed no binding to IL-24, indicating high selectivity for its target (Fig. S7). Our other promising IL-20 aptamers showed little to modest binding, with 10DA18 binding more than 10DB13 (Fig. S7). While high selectivity is the usual goal for aptamer identification, this cross-reactivity could be beneficial in certain applications.

**Fig. 6:**
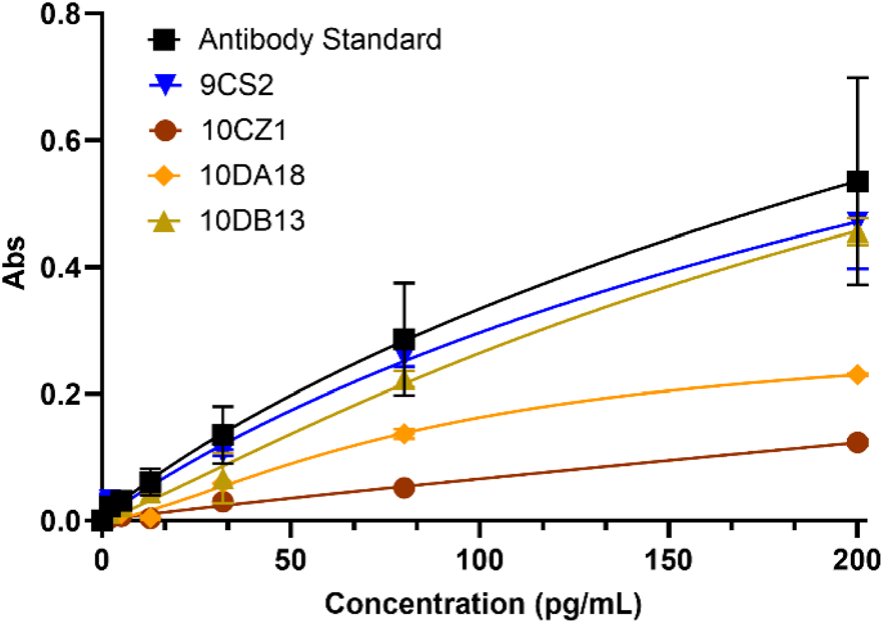
IL-20 aptamers compete with IL-20 antibodies for binding in a sandwich ELISA. Aptamers 9CS2, 10CZ1, 10DA18, and 10DB13 were added to increasing concentrations of IL-20 and allowed to bind. The complexes were then introduced to immobilized capture antibodies, followed by washing and addition of a biotinylated detection antibody and streptavidin-HRP. TMB was added to generate a colorimetric response that could be measured at 450 nm. The antibody standard curve was generated as described for the kit (Invitrogen). Error bars represent the standard deviation for *N* = 2 trials.

Finally, we tested the ability of our IL-20 aptamers to compete with anti-IL-20 antibodies in an ELISA assay (Fig. 6). In this assay, aptamers 10CZ1, 10DA18, and 10DB13 were introduced with IL-20 in a sandwich ELISA. If the aptamers are binding IL-20 in a region similar to the antibody, we anticipate a decrease in the absorbance from the coupled horseradish peroxidase (HRP) reaction. Our best binding IL-20 aptamer, 10CZ10, caused the largest decrease in activity (Fig. 6). Aptamer 10DA18 also showed lower activity, indicating its binding likely occurs in a region similar to the IL-20 antibodies. Aptamer 10DB13 had almost no impact on the assay, similar to our IL-17 aptamer 9CS2 which served as a non-binding control. These results further confirm the binding of our aptamers to IL-20, particularly 10CZ10 and 10DA18.

## Experimental

Plasmids containing the genes for human IL-17A and IL-20 were obtained from GeneCopecia (Rockville, MD, USA). DH5α and T7 express cells were obtained from New England Biolabs (NEB, Ipswich, MA, USA). Bacterial cell lysis buffer was purchased from Gold Biotechnology (St. Louis, MO, USA). Sulfo-SANPAH (sulfosuccinimidyl-6-(4′-azido-2′-nitrophenylamino)hexanoate), sodium acetate, piperidine, and Dynabeads M-270 carboxylic acid were from ThermoFisher (Waltham, MA, USA). DNA oligonucleotides were synthesized by Integrated DNA Technologies (IDT, Coralville, IA, USA) and purified either *via* denaturing polyacrylamide gel electrophoresis (PAGE) or phenol : chloroform : isoamyl alcohol (PCI) extraction. Amicon ultra 15K centrifugal filter units, phenol : chloroform : isoamyl alcohol 25 : 24 : 1, and Sephacryl S-100HR were from Millipore Sigma (St. Louis, MO, USA). Tetramethyl rhodamine (TAMRA)-NHS ester and BODIPY-TAMRA-NHS were purchased from Lumiprobe (Hunt Valley, MD, USA). PrepEase Ni resin was purchased from USB Corporation (Cleveland, OH, USA). Mouse IL17/17A and human recombinant IL-24 were from Sino Biological (Houston, Texas, USA). Human IL-17A/IL-17RA Inhibitor Screening Kit (TR-FRET) was purchased from Acro Biosystems (Newark, DE, USA). Human IL-20 ELISA kit was from Invitrogen (Frederick, MD, USA).

### Expression of IL-17 and IL-20

His-tagged IL-17 and IL-20 were expressed by transforming T7-express cells with appropriate plasmids using the manufacturer's protocol. Individual colonies were cultured overnight in LB media with ampicillin. Overnight cultures were used to inoculate 80 mL of LB media for expression. Cultures were incubated at 37 °C until the OD_600nm_ reached 0.3–0.5. Cultures were induced with 1 mM IPTG and were incubated for an additional 3 h at 37 °C. The cells were pelleted and lysed with bacterial cell lysis buffer with 1 mg mL^−1^ lysozyme. Cell debris and inclusion bodies containing the expressed interleukin were pelleted and the supernatant removed. Inclusion bodies were solubilized in 20 mM Tris pH 8.5, 6 M guanidine HCl, 100 mM NaCl, 5 mM EDTA, and 10 mM DTT for 30 min at room temperature and the resulting supernatant was collected for refolding. Isolated protein was refolded in 20 mM Tris pH 9.0, 500 mM arginine, 500 mM guanidine, 1 mM cysteamine, 5 mM cysteine, and 15% glycerol by mixing at 100 rpm for 40 h at 4 °C.^16^ After passing through a 10 000 kDa molecular weight cut-off column, the cytokines were purified *via* affinity chromatography with PrepEase Ni resin and the purity of the cytokines confirmed *via* SDS-PAGE. The purified cytokines were stored in 1× PBS buffer.

### Conjugation of IL-17 and IL-20 to magnetic beads

IL-17 and IL-20 were conjugated to carboxylic acid-functionalized magnetic beads. Beads were washed three times with 1× PBS to prepare for conjugation, followed by three washes with 25 mM MES pH 5. Cytokines were then conjugated to the washed beads using 10 mM EDC in 25 mM MES pH 5 overnight at 4 °C. The amount of protein conjugated was determined by comparing *A*_280_ readings of the solution before and after conjugation.

### SELEX for IL-17 and IL-20 aptamers

SELEX was initiated by introducing a randomized DNA pool with the sequence 5′ GAACTAGATCGCAGC-N_70_-GGATCGAGGTAATCC 3′ to the immobilized cytokines on the magnetic beads. The pool consisted of a variable sequence region of 70 nucleotides flanked by constant primer regions for PCR amplification. Four different buffer conditions were used for each cytokine and are designated as selection CS, CT, CW, and CX for IL-17 and selections CY, CZ, DA, and DB for IL-20, based on our lab naming conventions (Table S1).

300 picomoles of the randomized pool was annealed in appropriate binding buffer (Table S1) by heating at 95 °C for 5 min and cooling to room temperature for 15 min. The annealed pool was added to the bead-immobilized cytokines and incubated at room temperature with gentle mixing on a nutator for 1 h. Unbound oligos were removed by three washes with appropriate binding buffer. For the first wash, the beads were incubated in buffer for 30 min and incubation time was decreased to 5 min for the subsequent two washes. Bound oligonucleotides were eluted by addition of 200 µL of appropriate elution buffer (Table S1), which was repeated for a total of two elutions.

The eluted oligonucleotides were recovered *via* ethanol precipitation and were amplified using PCR. Ten cycles of PCR were followed by 30 cycles of PCR. A 5′-TAMRA-labeled forward primer at a ratio of 1 : 24 with an unlabeled forward primer was used during the 30 cycle PCR to add a fluorescent tag to the desired isolated sequence strand. This labeled primer was generated by conjugating TAMRA-NHS to a 5′ amine-modified forward primer (5′-GAACTAGATCGCAGC-3′). The reverse primer, 5′-CAACAACAACAAXGGATTACCTCGATCC-3′ contains a non-amplifiable linker (designated X) that allows strand separation on denaturing PAGE to regenerate the desired single-stranded pool. The desired single-stranded DNA product was isolated *via* denaturing PAGE and extracted from the gel as previously described for use in the following selection round (Fig. 2).^21^ The selection process was monitored starting in round 2 by measuring the fluorescence of the collected washes and elutions using a Spectra Max iD3 plate reader (Molecular Devices, San Jose, CA, USA). The excitation wavelength was set at 540 nm, and the emission was measured at 590 nm at a scan rate of 4 ms.

To isolate highly specific aptamers, counter selection steps were conducted to remove nonspecific binders. Two negative selection steps were incorporated. For the first counter selection, conducted at round 3 for IL-17 and round 5 for IL-20, enriched pool sequences were introduced to functionalized magnetic beads without conjugated cytokines. Non-binding sequences removed during the wash step were then introduced to the conjugated cytokines, with subsequent washing and elution as described above. For the second negative selection, a 5× concentration of IL-20 with 1% Bovine Serum Albumin (BSA) solution was added to the wash step for IL-17. Similarly, a 5× concentration of IL-17 in 1% BSA solution was added to the wash step for IL-20. The 5× concentration was relative to the concentration of immobilized cytokine on the beads. IL-17 and IL-20 are similar in size with some sequence homology, and bovine serum albumin (BSA) is used as a crowding agent to stabilize cytokines for biological applications. Elution steps then were conducted as described above.

Once increased elution was observed, the recovered oligonucleotides from round 9 for IL-17 and round 10 for IL-20 were cloned as previously described.^21^ The resulting sequences were analysed for duplicates and aligned using T-coffee.^22,23^ Alignments were visualized using N·ESPript.^24^

### Characterization of identified aptamers

The unique aptamers were tested with a method similar to SELEX. TAMRA-labeled aptamers were generated from plasmid templates *via* PCR using the forward and reverse primers from SELEX. The aptamers were then annealed in appropriate binding buffer as described and incubated with their immobilized cytokine target for 1 h at room temperature with gentle mixing on a nutator. The beads were then washed twice with binding buffer, followed by two elutions, as previously described. Fluorescence measurements were taken for the combined washes and the combined elutions for each aptamer (Tables S5 and S6). The aptamers with the highest percentage elution in each SELEX (1 to 3 aptamers) were ordered as 5′ amine-modified oligonucleotides for further testing using fluorescence polarization to determine binding affinity (Table S7).

### Fluorescence polarization

Bodipy-TAMRA NHS was conjugated to 5′ amine-modified aptamers for fluorescence polarization assays. Annealed aptamer was incubated with increasing concentration of target cytokine in appropriate binding buffer for 1 h at room temperature. Samples were then transferred to a 96 well plate for fluorescence polarization measurements with excitation at 542 nm and emission at 574 nm. *K*_d_ values were determined by plotting the resulting fluorescence data in GraphPad Prism (version 10.6.1) and using the non-linear fit function with the equation:*Y* = bottom + (*X*^Hillslope^) × (top − bottom)/(*X*^Hillslope^ + *K*^Hillslope^_d_)where bottom and top are the low and high polarization values, respectively, and Hillslope is the Hill coefficient.

### Photo crosslinking of aptamers with cytokines

The photo-crosslinking reagent sulfo-SANPAH was conjugated to 5′ amine-modified aptamers and a non-binding control oligonucleotide in CHES buffer pH 9.1 at 37 °C overnight, followed by ethanol precipitation. The conjugated aptamer was annealed as described above and added to an equal molar ratio of appropriate cytokine and allowed to bind for 1 h. Samples were cross-linked by exposure to 400 nm light for 15 min. The crosslinked complexes were analysed *via* 10% SDS-PAGE.

### IL-17A aptamer inhibitor assay

Assays were conducted using the Human IL-17A/IL-17RA Inhibitor Screening Kit (TR-FRET) from Acro Biosystems. The provided anti-IL-17RA neutralizing antibody was used as a standard control following the manufacturer's protocol. Tested aptamers were annealed as described and diluted to the same concentrations used for the standard control in a 96-well tray. Human IL-17A europium-chelate (donor) was added to each concentration of aptamer and allowed to bind for 30 min at room temperature. FA labelled human IL-17RA (acceptor) was then added to each well and allowed to bind for 30 min at room temperature. Fluorescence was measured by exciting at 337 nm and detecting the emission at 620 nm and 665 nm. The TR-FRET ratio equal to emission_665nm_/emission_620nm_. Data was plotted in GraphPad Prism using the non-linear fit EC50 shift.

### ELISA competition assay

Assays were conducted using the Human IL-20 ELISA kit from Invitrogen. The provided recombinant human IL-20 was used to create the standard curve and as the aptamer target. Tested aptamers with a fixed concentration of 170 µM were annealed as described and incubated with increasing concentrations of IL-20 for 1 h at room temperature. Complexes were introduced to human IL-20 antibody-coated wells and were incubated for 2.5 h at room temperature. The solution was removed and the wells washed four (4) times with provided wash buffer prior to incubation with the biotin-conjugated secondary antibody for 1 h at room temperature. Following washing, streptavidin-conjugated HRP was introduced and incubated for 45 min at room temperature. Wells were again washed and the TMB substrate was added and incubated for 30 min at room temperature. After addition of the stop solution, absorbance was measured at 450 nm. Data was plotted in GraphPad Prism using the non-linear fit specific binding with Hill slope-test.

## Conclusions

This study successfully identified DNA aptamers for both IL-17A and IL-20, two important cytokines implicated in diabetic wound healing and psoriasis. Using SELEX, we isolated high-affinity aptamers for each target. For IL-17, the aptamer 9CS2 demonstrated a strong binding affinity with a *K*_d_ of 4.16 nM. For IL-20, aptamer 10CZ1 showed promising binding with a *K*_d_ of 120.2 nM.

The binding of these aptamers to their respective targets was further confirmed through several assays. Photo-crosslinking experiments showed clear shifts in band migration for both 9CS2 with IL-17 and 10CZ1 with IL-20. Our IL-17A aptamers were specific for the human form used in SELEX, with little binding observed to mouse IL-17A. These aptamers were also able to interfere with IL-17A binding to its IL-17RA receptor in an inhibitor assay. Our best IL-20 aptamer, 10CZ1, was highly selective for IL-20 compared to the closely related IL-24, while our other promising IL-20 aptamers showed moderate selectivity. 10CZ1 and, to a lesser extent, 10DA18 were able to interfere with anti-IL-20 antibody binding in an ELISA assay.

These novel DNA aptamers for IL-17 and IL-20 represent valuable tools for potential therapeutic applications in diabetic wound healing and psoriasis treatment. Their high affinity, combined with the inherent advantages of aptamers over antibodies, make them promising candidates for further development as future studies will focus on evaluating the efficacy of these aptamers in modulating IL-17 and IL-20 activity, as well as exploring their potential in combination therapies for enhanced treatment outcomes in diabetic wound healing and psoriasis.

## Author contributions

AP was responsible for conceptualization, data curation, formal analysis, investigation, methodology, resources, visualization, and writing. KS was responsible for investigation. DAB was responsible for conceptualization, data curation, formal analysis, methodology, resources, supervision, visualization, and writing.

## Conflicts of interest

There are no conflicts to declare.

## Supplementary Material

RA-016-D6RA00178E-s001

## Data Availability

The data supporting this article have been included as part of the supplementary information (SI). Supplementary information: additional experimental details, sequencing results and analysis, initial binding studies, fluorescence polarization results for *K*_d_ determination, DMS probing details and analysis. See DOI: https://doi.org/10.1039/d6ra00178e.
