# Supplementary material for: DNA aptamers that inhibit binding to human interleukin-17A and interleukin-20
Source: RSC Adv. 2026 Apr 20;16(22):20340–6. doi: 10.1039/d6ra00178e (PMC13093883; doi:10.1039/d6ra00178e)
Supplement: RA-016-D6RA00178E-s001 [file RA-016-D6RA00178E-s001.pdf]

Supplementary Information (SI) for RSC Advances.
This journal is © The Royal Society of Chemistry 2026

Supplemental Information for

DNA Aptamers That Inhibit Binding to Human Interleukin-17A and Interleukin-20

Ali Parvez, [a] Kirsten Sully [a] and Dana A. Baum *[a]

Department of Chemistry

Saint Louis University

3501 Laclede Avenue

St. Louis, MO, USA

dana.baum@slu.edu

Table of Contents

Supplemental Experimental - Dimethyl Sulfate (DMS) probing....1
Supplemental Discussion - DMS Probing Analysis....1
Table S1. Buffers used during SELEX....2
Table S2. Sequencing results for cloning of IL-17 Aptamer Selections CS and CT....3
Table S3. Sequencing results for cloning of IL-20 Aptamer Selections CY and CZ....4
Table S4. Sequencing results for cloning of IL-20 Aptamer Selections DA and DB....5
Table S5. Initial binding assays for IL-17 aptamer sequences....6
Table S6. Initial binding assays for IL-20 aptamer sequences....7
Table S7. Oligonucleotides used for fluorescence polarization studies....8
Table S8. K_d values for aptamers determined by fluorescence polarization....9
Figure S1. Sequence alignment for 9CS and 9CT clones....10
Figure S2. Sequence alignment for 10CY, 10CZ, 10DA and 10DB clones....11
Figure S3. Fluorescence polarization of IL-17 binding aptamers....12
Figure S4. Fluorescence polarization of IL-20 binding aptamers....13
Figure S5. Fluorescence polarization of IL-17 binding aptamers with mouse IL-17....14
Figure S6. DMS probing of aptamer 10CZ1....15
Figure S7. Fluorescence polarization of aptamers with IL-24....16

Supplemental Experimental - Dimethyl Sulfate (DMS) probing

5’ TAMRA-labeled 10CZ1 was incubated with increasing concentrations of IL-20 in appropriate binding buffer for 1 h at room temperature. 200 uL of the resulting solution was then treated with 5 uL of 10% v/v DMS at room temperature for 5 min. Reactions were quenched with 50 uL of a stop solution containing 1.5 M sodium acetate (pH 7.0) and 1 M beta-mercaptoethanol. Following ethanol precipitation, the methylated aptamers were treated with 1 M piperdine at 95 °C for 10 min. The resulting cleavage products were analyzed via denaturing PAGE. Changes in cleavage patterns were determined by quantifying band intensities using Image Lab (Bio-Rad, Hercules, CA, USA) and comparing to the band intensities for cleavage of the folded aptamer without added IL-20.

Supplemental Discussion - DMS Probing Analysis

In comparing the DMS cleavage patterns between folded 10CZ1 in the presence and absence of its IL-20 target, we observed pattern differences were localized to nucleotides 1 – 40 and focused our analysis on this region of the aptamer. Secondary structure prediction (shown in Figure S5) indicates nucleotides 41 – 100 can form a rather stable structure dominated an extended paired region with two loops. In future work, this region can likely be shortened. Our probing studies indicate that upon binding of IL-20, the region of the aptamer from A6 (As are also susceptible to DMS modification and cleavage, but to a lesser extent than Gs) to approximately G20 become less prone to cleavage, indicating protection from IL-20 binding. This is concurrent with the region containing G26 to approximately G37 becoming more prone to cleavage. We hypothesize that IL-20 binding disrupts the proposed structure and opens G26 – G37, with IL-20 interactions occurring with A6 – G20.

Table S1. Buffers used during SELEX

Selection Buffer	Composition
Binding buffer CS/CY	10 mM $CaCl_2$, 1X PBS pH 7.4 , 0.02% Tween 20
Binding buffer CT/CZ	10 mM $MgCl_2$,1X PBS pH 7.4 , 0.02% Tween 20
Binding Buffer CW/DA	10 mM $CaCl_2$, 20 mM Tris pH 7.4, 5 mM KCl, 0.02% Tween 20
Binding Buffer CX/DB	10 mM $MgCl_2$, 20 mM Tris pH 7.4, 5 mM KCl, 0.02% Tween 20
PBS Elution Buffer	1X PBS pH 7.4, 3.5 M urea, 10 mM EDTA, 0.02% Tween 20
Tris Elution Buffer	20 mM Tris pH 7.4, 3.5 M urea,10 mM EDTA, 0.02% Tween 20

Table S2. Sequencing results for cloning of IL-17 Aptamer Selections CS and CT

	GAACTAGATCGCAGC – Random Region – GGATCGAGGTAATCC
9CS2	CCAACCGGTGACGCACAGTTAGCAACGAATCCCCTAGCCTCGATATGACAGTCATACCCGTCGCATGTGG
9CS3[a]	CTAATGCCCGTACATAGCCACGCATACTGTTTTTCTAATAAAGCTCCTTGAGGTGAACCGTCGCTGGGTG
9CS5[b]	CCACAGCATCACCGTCTATCCGGCAGTGACACTTGAACTTGCCCTTTTCCATCTTGTG
9CS6	CCCACGAGGGTTTATCTCCTACGTGCAACAAGTCCACTATAGGATCAGAGATGAGTTTGGTCGTGCAGTG
9CS9[c]	CCACGGGAGACGCATTAAGCCGATTCACTATCCCTACCCGTGGGACCGATCTAGGACCGGTCAGTGTGTG
9CS11	CCACCAACCAAGGACCATAAGCAGGTCCAACTAGGGCGGGCGATAGACAGCGGCACTGTTGTCAGTTGGG
9CS12	CCCAGCAGGTTAAAGTGTAAGTAGCAACCAAAAGGATACGATGTTTCTGGTAGTCCCTCGCCTCTCGGTG
9CS14a[d]	CCACACACAATAGTCCGCTGCCACGAACACGACCTAATGTCGTTCATAACGATTATATGTAGCTCCGTGG
9CS18	CCCACGCACCGCGGCCTCACTGACATTATCTCCTAAACCAGTTGACTAAAGCACCGTGCATTCACCTTGG
9CS19b[d]	CCACGGCGGGACCCGCTAGACGGCAGGGGACTCTTGAACTATCCGTCTGACGCGTGTGGGG
9CT1[e]	CTAATGCCCGTACATAGCCACGCATACTGTTTTTCTAATAAAGCTCCTTGAGGTGAACCGTCGCTGGGTG
9CT2	CCCCCGGGTCACCGTTATAATCCGCTATCCAAAACCCGATTGACGTACTGCTATTGAAGCCGGGAGCGTG
9CT3[b,f]	CCACAGCATCACCGTCTATCCGGCAGTGACACTTGAACTTGCCCTTTTCCATCTTGTG
9CT6	CCCAGGGACGAGTATGGCGACATGGGATGGCACTAGTCGGTTGTCCAAACACGTTGATTTGTCCCCGGTG
9CT11	CCAAGGTGTTAAGAGGCGTGTGCTGGAAACTCGTTCCATCACCGGAGGTTTTGACAAGACACTCTGTTGG
9CT12	CACATCATATGAGTCCCATTCGGCAGTGACTCTTGAACGCCGGGTCTTGATTGTGCTG
9CT13	CCACGGGAGACGCATTAAGCCGATTCACTATCCCTACCCGTGGGACCGATCTAGGACCGGTCAGTGTGTG
9CT17	CCACAGCATCACCGTTATCCGGAGTGACACTTGAACTTGCCCTTTTCCATCTTGTG
9CT18	CCACAGTCACGACATCGCTTCGTGACATCACATACGTGATTTCACTACAATTGCTCCGTTTACCCTTGTG
9CT19	CCACCGACACACACCGTATATCCCCTAGCCCCACATGAGGCCATCTCGCAACTGGACGTTCGCTTCTGGG
9CT20	CCTGTACATAGCCACGCATACTGTTTTTCTAATAAAGCTCCTTGAGGTGAACCGTCGCTGGGTG
9CT21	CACACCAGTATAGGCCACAGTTGTGCTATACGAGATAAACCTATGCCACATTCCAACGTCTTCGCGTGTGG

[a]Sequence was found in 10 additional clones (9CS4, 10, 13, 14b, 15, 16, 19a, 20, 21, and 22)

[b]Sequence found in CS and CT clones

[c]Sequence found in 1 additional clone (9CS17)

[d]Plasmid contained more than one insert with different sequences, designated a and b.

[e]Sequence found in 1 additional clone (9CT16)

[f]Sequence found in 3 additional clones (9CT7, 14, and 15)

Table S3. Sequencing results for cloning of IL-20 Aptamer Selections CY and CZ

	GAACTAGATCGCAGC – Random Region – GGATCGAGGTAATCC
10CY1	CACCGGCGGAAGTGCGAAATCTGGGTTACGCAGGCACTCCTACGTCTCTCTTCCCGACTGCGTTCGAGGTAACTTTGG
10CY2	CACACACGCCAGCCGACGACTCCACTTAGCTATACCAATATCTCAGCAAGTTAACGTACTACGCGGTCCC
10CY4	ACGACGCGCTTACTTTTTACGATTATGAATGCCGCTAAATACTCCGCTGTCGCGTACTGCGGAAGCGCCCG
10CY5	CGCGGGAATAGTGGCTAATACCGGGTGTTATGCGACTGATGTATGCCCCGTGTACGGAAGGTCGGCGGTTGTG
10CY6	ACGACGCGCTTACTTTTTACGATTATGAATGCCGCTAAATACTCCGCTGTCGCGTACTGCAAAGCGCCCA
10CY7	CACGGGAATAGGGGCTAATTCCGGGTGGAAGGCACTCAATTTGCCCCACGTACAAAGGTCGGCGGTCC
10CY8	ACCCATTGGAGTAATCCACAGAAATTCCCGGGCCACCTATTATTCGGAGCCGAACACTTCTTCCTTGCGT
10CY9	CACCCACAATCGATAAGATCTTCACCTACCGACAAAGCGGTACACCAGGTATCTTGCCCTCTGCCC
10CY10	CTAATGCCCGTACATAGCCACGCATACTGTTTTTCTAATAAAGCTCCTTGAGGTGAACCGTCGCTGGGTG
10CY11	ACCACGGCCGACCATGTACAGGCGATAAACCCTTCAATGTCACCTACGAGGACCGGGAACTACTAATATG
10CY12	CCCCACAGTGTGATCTAACCCGCCGCTTCCAGGAACCACATAGATGATAAGAGTTTCCCGCTGCCCGCCC
10CY13	CTAATGCCGTACATAGCCACGCATACTGTTTTTCTAATAAAGCTCCTTGAGGTGAACCGTCGCTGGGTG
10CY15	CCCACAGCAGCCCTTGTTCACTGGGGACGAGTCAAGAATTGAAGGCCTGAGTCAGTTCCCTATCCGTGTG
10CY16	CCACGACACTATTCTAGCAACTAAATGACCGACCTAAACAACCGTGTGGAGCCGTTCTCCTTCCGATATG
10CY17	CCACGGGAGACGCATTAAGCCGATTCACTATCCCTACCCGTGGGACCGATCTAGGACCGGTCAGTGTGTG
10CY18[a]	ACGACGTTTCCTTATACTTATCCTCCCCGGGTAGTGATGCTTACCGTTCAGAGGATTGTTGGGCCCACGT
10CY19	CACACCCCGACTCCCCAGATTGAAGTCTGTAGTATGTTATGACAGTGTAAATCGTCTCATGGTTAGCCGT
10CZ1	GGCAGCACACGTCCAGTGTAGGCGTCATCCCTCTATCCAATCGTCGTTCACAGCATTATCATGGGTTGTG
10CZ2	CCAACACTCAGTAATGGTACGTAGCGGCACGCTAAGTGAGTAAGTACATTAGCTGTTTACCTCCAGTGTG
10CZ4	CTAACGCCCGTACATAGCCACGCATACTGTTTTTCTAATAAAGCTCCTCGAGGTGAACCGTCGCTGGGTG
10CZ6	CATGTGGGTTTTCTCTGAAGCAACCCATCCGATCTACCTATAATTCTTTGACTAAATGAAGTTCGCCCGG
10CZ7	CAGAACGTGGCTCGTGCTTAGTTCTACCAGTGGAATGTGAGATTATAGTACCGTCTACTTGGCCGTCCC
10CZ8	CTAATGCCCGTACATAGCCACGCATACTGTTTTTCTAATAAAGCTCCTTGAGGTGAACCGTCGCTGGGTG
10CZ9	AGGGCAAAAATAGGTTTATCCGTAAGTCAACTAAGTTATAATTTCTCCGTTAGTAGGTGGGGAACTTTCA
10CZ10	CTAATGCCCGTACATAGCCACGCATACTAATAAAGCTCCTTGAGGTGAACCGTCGCTGGGTG
10CZ11	CCACAGGCTCAACATTCAATCCGAACGAAACCTTCCTAACTAGGTCATCTCTTGGCGTCGAACTGCGGCC
10CZ12	CAAGCGGTGACCATGTTGGTTGGAGCTTCTTACCGCGTGTTACCACTGTGTATCTGGTCTGGAGCATGG
10CZ13	CTACCCCGTCGTGGAGAGTTTTTTCAGCATGGTTACACAATACGTATACAGCTTCCCCAGCGCGGTTGCC
10CZ14[b]	CAGAAGACAGCATTTAGTACATTGGAGCAGTCGGCCAGTTATGTAGATTCAACCATCAGTAAAACGCCTC
10CZ16	ACCACACTACAACGTAGCCTACAGGGCCCAAAGTATAGCCATGCCGTCCGATTCCGTCGTGCCTTACCCC
10CZ17	CAGCAGGTATAGGTATCACTGACAGCTATTCGCGCACTAAGGAAGTTACAGCATTTATATGACGCACCCC
10CZ18	CACACCCGAGGGCCTACAAATGAGACAGGGTGGCAATATCCAGAACTGTGGTGATACATTGCAGCCTAGG
10CZ20	CCACGGGAGACGCATTAAGCCGATTCACTATCCCTACCCGTGGGACCGATCTAGGACCGGTCAGTGTGTG

[a]Sequence was found in 1 additional clone (10CY20)

[b]Sequence was found in 1 additional clone (10CZ19)

Table S4. Sequencing results for cloning of IL-20 Aptamer Selections DA and DB

	GAACTAGATCGCAGC – Random Region – GGATCGAGGTAATCC
10DA1	ATGCACGCACATGATAAAGCCCGAGGACGTATTAATGATGTGGAACCGGGAATTTGTTACAACGGCTGGG
10DA4	CCGCGCTCTCCCGATCGGCGCCGTGAATAACGACGAAACCCGAGTCTTTGGGTCGAGACGTTAAGCTCGGTTCTAT
10DA6	CCGCGGATCGCTTTTGGCTAGCTGTAGCTCCGCGCGAACAGCGTCTGCTTAGGGGATCTAGGCCGGGGTG
10DA7	CACCACAGTTACCGATCGACATTCCACTTGACACTCCCCCTGTATCCCGTGTGCTCCACTCGTCCTGGTG
10DA9	CACAGCACGTCCACCAGACATAAATAGTCACCAAGGAGGTCCATACGATTCGCAAGCCCAGCATTGTCCG
10DA10	CTCCCCTGGACCTAGGGATTGCGAGGTGAATGATCTGTAGTAGCTCGTACGACGCGAGCTTATTGGCGTGA
10DA11	CACGACACCTACCGACACCTTATCTCACCGTTTATTCTGATCAGCTCTCAACCAGCATGCCCGGCTATCG
10DA13	CACCAGGGTATGTAAACGGCGAGCTGAGATATCAACGGACAATTAAACATTGATGCGACTCACCCATGCA
10DA14	CACAACTACGTGATCAACGAGGAAATATAAGCAAATAGACGGAATTCCAAACTAAATCAGACTTAGTCCC
10DA16	CACAGCATCGAGGGGGTAAGACCCCACGTCACAGTAACTCATGGAACTTCACACAATGATCTCCCGTCCC
10DA17	GAGCAAAGTCTAAGAGTTTCACCATGGCGGACCCCCACTCACTACTCGAACTATTAACCCGCAGCGTATTCTCCTGCC
10DA18	CCACCCGCATAGCGCAGCATACGAACCCCCGAGGATCGATACAACGGGCTACGACATAAAGTACTATTAG
10DB1	CGGCACGAACACGATGTTCCTCGTTAGTTTATTTAGTCCGACACGAGGGCTCGCCCCGTACTATGGTCCAGTG
10DB2	CGGCACGAACACGATGTTCCTCATTAGTTTATTTAGTCCACACAGGGCTCACCCCGTACTATGGTCAGTG
10DB5[a]	CACACAGAACAACTGAATACGAGGGCCGGTCCCGTATTAAATCCCTCGAGTGCTGTAGATCACGATTCAC
10DB6	CTACCGACAACGCACCGTCAACCAATGAATTATCCGATTCCATTCCACCTCTTTGTAGACGACGTCCCCC
10DB7	CCAGCGCGCGGCGAACGTCATGTAAGTGTTTCCAATTCTACTAACGGATATACTTTCGTATACGGACCAG
10DB8	CCAACCGGGTAACTAATCATGAAGAATTCGAATGCTATAACGGAGAGAGTCTGCCGATCACGTCGTGCTT
10DB9	ACGCAGTTACACCTCACATATTGTTCAAACTTCCCACATCGTTCACCAGAAACTCGCCAGTTCCCGGCAT
10DB10	TATACTCGCCTACGAAAAAGTAAATTTCATCACCACTTACGATTCGGGCGAACCTCACGTACATTGGGGC
10DB13	CAAACGCCACGATACCCACTATAAGTCGCCGGCCTCCCAATCAACGCCGCACGCACGCTCTATACTCCCCAG
10DB14	CCACACCTACTACAGCCGTCTAAAGACACACATGGGTCGAAATTCAGACACCGGGCGCCACCCCTGCGGCGGGC
10DB15	CCACACACTATGAACCTAGCGGCCGTGTCTATTACGGTGCCCGGAGCTCTCTCGTACTACGGACTGCCCGAGTCCTCA
10DB16	CAGTAACCTGCATTGTGAAACTAAACATGCCGAGCATAGCGGATACTGTGGTCACTGTTGCCACAGTAGG
10DB17	GGAACCGGCCACCCAAATCGATTGGAAGGTCAGATCTGTGGAGGAATATGAGGGCTGCGAACGCAATAG
10DB18	CGAGCTCCCTAGTGCACATCTACACCATGTCAATACGACCTCTGCGTGTTAGTTAATAAATGATCGACTA
10DB19	ACCACGACCGGCAGTGTACATCGGAGCTTAATAAGCTCTCTTCGAATGGTAATTACTATTACGTCAGTAC
10DB20	CAGCCGTCGACAAACCTTCTCCTGTAAACTCCCCGGTCTAGTTCCTAGACACTTCGCCCATTAGCTAGTG

[a]Sequence found in 1 additional clone (10DB11)

Table S5. Initial binding assays for IL-17 aptamer sequences

Sequence	Wash	Elution
9CS2	4	96
9CS3	7	93
9CS5	2	98
9CS6	6	94
9CS9	5	95
9CS11	6	94
9CS12	7	93
9CS14a	13	87
9CS18	2	98
9CS19b	10	90
9CT1	24	76
9CT2	37	63
9CT3	38	62
9CT6	31	69
9CT11	45	55
9CT12	15	85
9CT13	31	69
9CT17	45	55
9CT18	43	57
9CT19	30	70
9CT20	51	49
9CT21	46	54

Table S6. Initial binding assays for IL-20 aptamer sequences

Sequence[a]	Wash	Elution
10CY2	57	43
10CY5	24	76
10CY6	29	71
10CY7	21	79
10CY9	31	69
10CY10	43	57
10CY11	43	57
10CY12	35	65
10CY13	39	61
10CY16	27	73
10CY17	32	68
10CY18	30	70
10CY19	79	21
10CZ1	16	84
10CZ4	46	54
10CZ6	24	76
10CZ8	33	67
10CZ10	43	57
10CZ12	49	51
10CZ13	31	69
10CZ14	60	40
10CZ16	53	47
10CZ17	45	55
10DA1	78	22
10DA6	23	77
10DA7	5	95
10DA10	31	69
10DA11	24	76
10DA14	21	79
10DA16	26	74
10DA17	39	61
10DA18	17	83
10DB1	32	68
10DB7	51	49
10DB8	65	35
10DB10	40	60
10DB13	23	77
10DB14	58	42
10DB15	33	67
10DB16	36	64
10DB17	29	71
10DB18	32	68

[a]Clones that were sequenced, but not included in table failed to produce sufficient PCR product for testing.

Table S7. Oligonucleotides used for fluorescence polarization studies

	Sequence
9CS2	GAACTAGATCGCAGCCCAACCGGTGACGCACAGTTAGCAACGAATCCCCTAGCCTCGATATGACAGTCATACCCGTCGCATGTGGGGATCGAGGTAATCC
9CS3	GAACTAGATCGCAGCCTAATGCCCGTACATAGCCACGCATACTGTTTTTCTAATAAAGCTCCTTGAGGTGAACCGTCGCTGGGTGGGATCGAGGTAATCC
9CT6	GAACTAGATCGCAGCCCCAGGGACGAGTATGGCGACATGGGATGGCACTAGTCGGTTGTCCAAACACGTTGATTTGTCCCCGGTGGGATCGAGGTAATCC
9CT19	GAACTAGATCGCAGCCCACCGACACACACCGTATATCCCCTAGCCCCACATGAGGCCATCTCGCAACTGGACGTTCGCTTCTGGGGGATCGAGGTAATCC
9CT20	GAACTAGATCGCAGCCCTGTACATAGCCACGCATACTGTTTTTCTAATAAAGCTCCTTGAGGTGAACCGTCGCTGGGTGGGATCGAGGTAATCC
9CT21	GAACTAGATCGCAGCCACACCAGTATAGGCCACAGTTGTGCTATACGAGATAAACCTATGCCACATTCCAACGTCTTCGCGTGTGGGGATCGAGGTAATCC
Aptamer 1 from Ref 15	GCTGTGTGACTCCTGCAACTGGATTGTATGATCTGTCAGGAGCACCGTCTGAGGGTACACACGCCTATGACCTGTGCAGCTGTATCTTGTCTCC
Truncated Aptamer 1 from Ref 15	ATACAACTGGATTGTAT
Aptamer 2 from Ref 15	GCTGTGTGACTCCTGCAATTAACTGTTTTCTTTTGTTTTTATGTTGTTGGCTTTTTCTTTTGCAGCTGTATCTTGTCTCC
Truncated Aptamer 2 from Ref 15	CGACTAACTGTTTTCTTTTGTTTTTAGTCG
10CY7	GAACTAGATCGCAGCCACGGGAATAGGGGCTAATTCCGGGTGGAAGGCACTCAATTTGCCCCACGTACAAAGGTCGGCGGTCCGGATCGAGGTAATCC
10CZ1	GAACTAGATCGCAGCGGCAGCACACGTCCAGTGTAGGCGTCATCCCTCTATCCAATCGTCGTTCACAGCATTATCATGGGTTGTGGGATCGAGGTAATCC
10DA7	GAACTAGATCGCAGCCACCACAGTTACCGATCGACATTCCACTTGACACTCCCCCTGTATCCCGTGTGCTCCACTCGTCCTGGTGGGATCGAGGTAATCC
10DA14	GAACTAGATCGCAGCCACAACTACGTGATCAACGAGGAAATATAAGCAAATAGACGGAATTCCAAACTAAATCAGACTTAGTCCCGGATCGAGGTAATCC
10DA18	GAACTAGATCGCAGCCCACCCGCATAGCGCAGCATACGAACCCCCGAGGATCGATACAACGGGCTACGACATAAAGTACTATTAGGGATCGAGGTAATCC
10DB13	GAACTAGATCGCAGCCAAACGCCACGATACCCACTATAAGTCGCCGGCCTCCCAATCAACGCCGCACGCACGCTCTATACTCCCCAGGGATCGAGGTAATCC

Table S8. K_d values for aptamers determined by fluorescence polarization

Aptamer	Target	K_d
9CS2	IL-17	4.2 nM
9CS3	IL-17	9.9 nM
9CS6	IL-17	ND[a]
9CS9	IL-17	ND
9CS18	IL-17	ND
9CS19b	IL-17	ND
9CT3	IL-17	ND
9CT6	IL-17	12.5 nM
9CT11	IL-17	ND
9CT19	IL-17	5.3 nM
Aptamer 1 from Ref 15	IL-17	ND
Truncated Aptamer 1 from Ref 15	IL-17	ND
Aptamer 2 from Ref 15	IL-17	3.1 nM
Truncated Aptamer 2 from Ref 15	IL-17	2.5 nM
10CY7	IL-20	0.3 uM
10CZ1	IL-20	0.1 uM
10DA7	IL-20	>1.3 uM
10DA14	IL-20	>22.0 uM
10DA18	IL-20	0.2 uM
10DB13	IL-20	1.0 uM

[a]ND = Not determined. In these cases, aptamer binding curves were not consistent with single binding events.

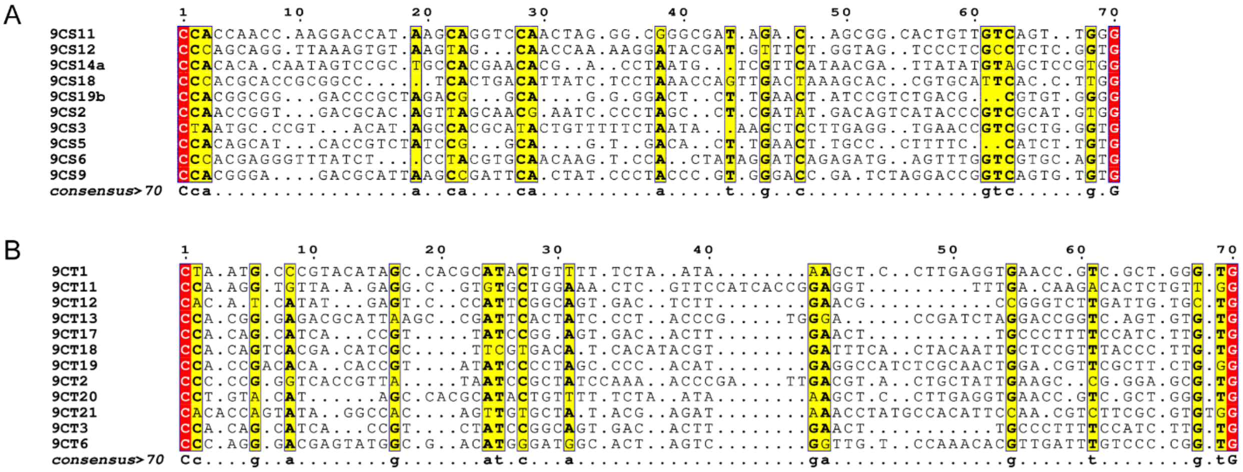

Figure S1. Sequence alignment for 9CS and 9CT clones. The unique sequence regions for the clones from selection 9CS (A) and 9CT (B) were aligned using T-Coffee[18,19] and displayed using N*ESPRIPT.[20] Duplicate clone sequences and the primer binding regions were not included in the alignments.

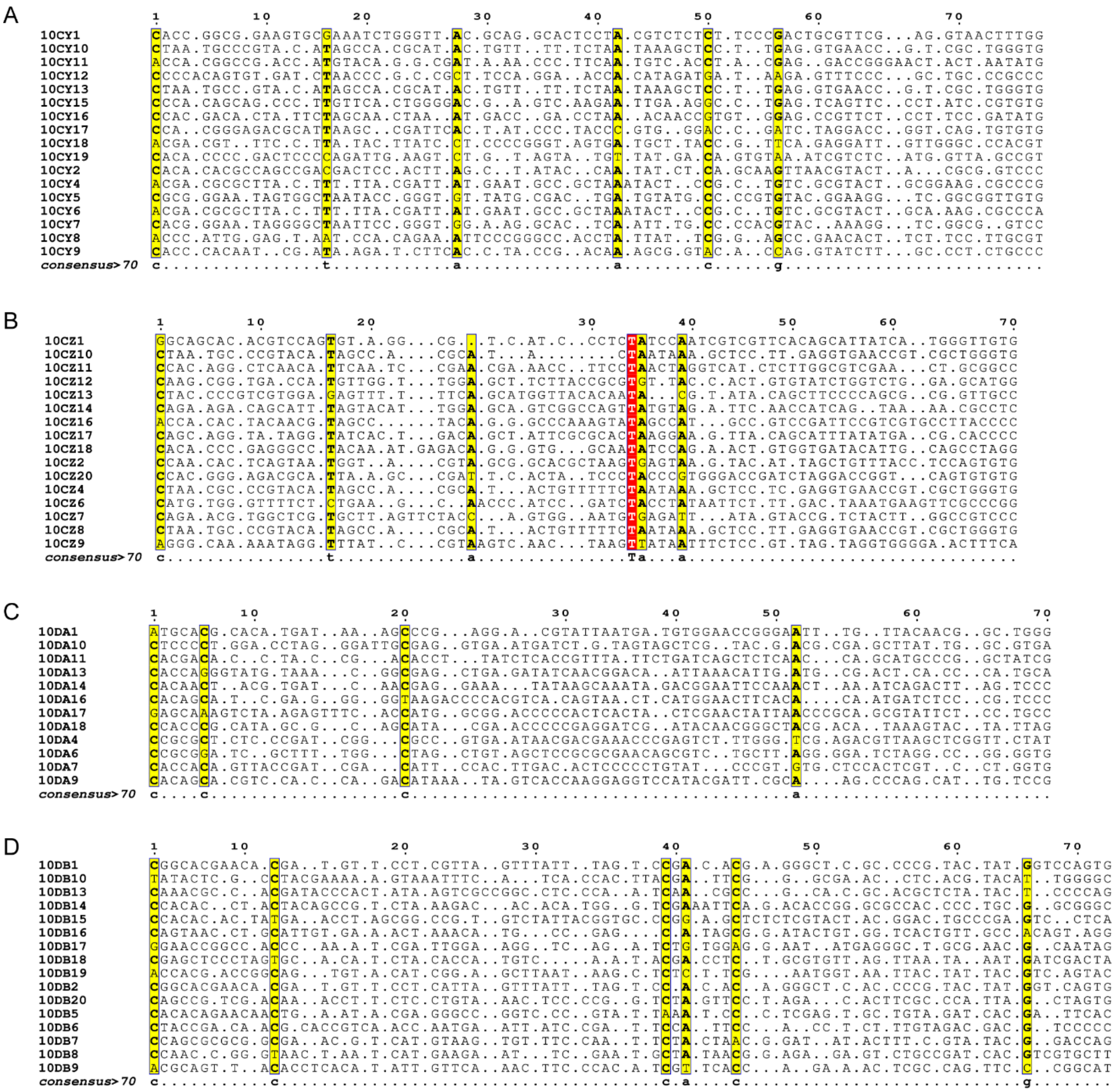

Figure S2. Sequence alignment for 10CY, 10CZ, 10DA and 10DB clones. The unique sequence regions of the clones sequenced from selection 10CY (A), 10CZ (B), 10DA (C) and 10DB (D) were aligned using T-Coffee[18,19] and displayed using N*ESPRIPT.[20] Duplicate clone sequences and the primer binding regions were not included in the alignments.

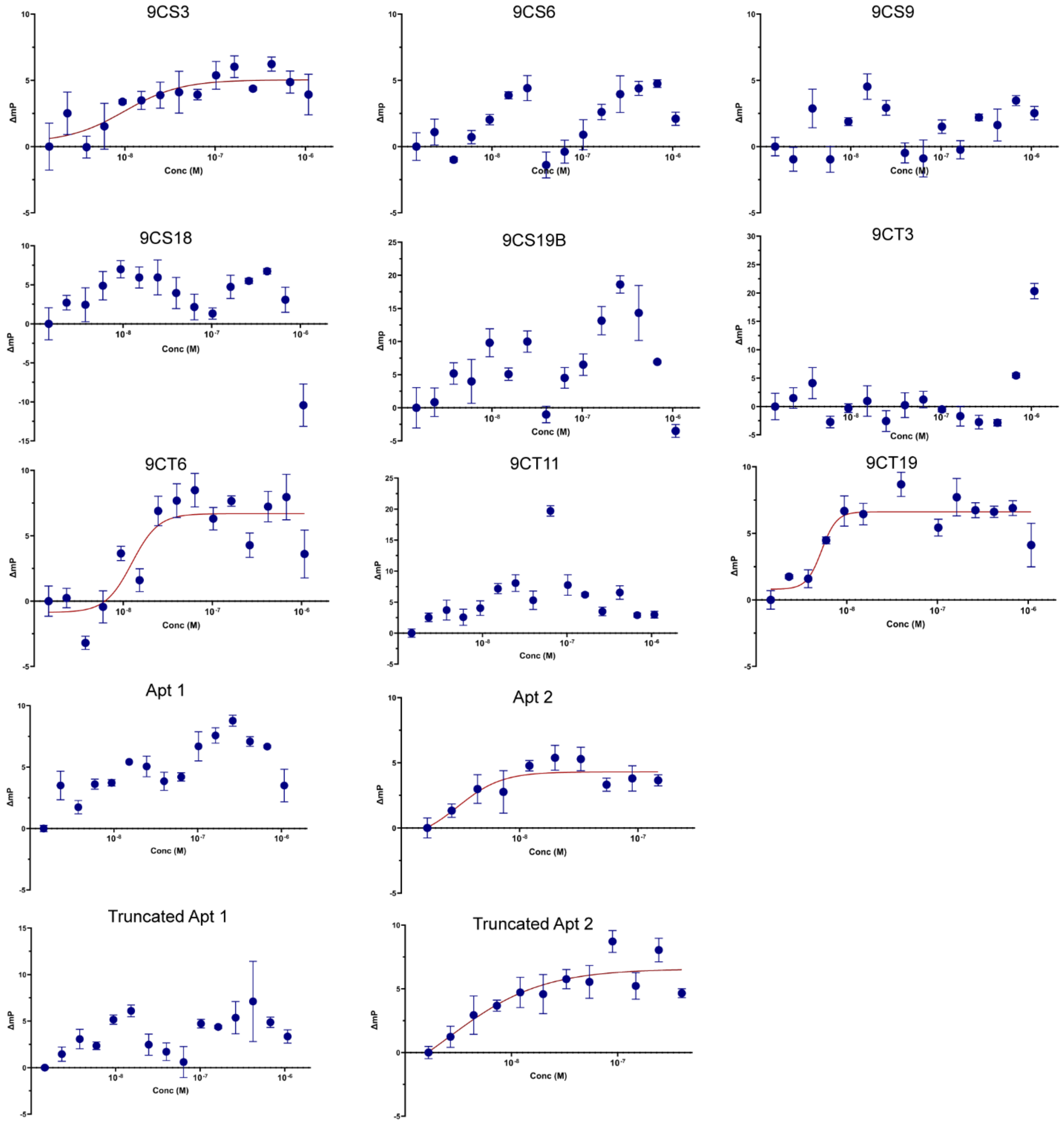

Figure S3. Fluorescence polarization of IL-17 binding aptamers. Binding constants were determined from non-linear fits using GraphPad Prism as described. Aptamers lacking curve fits appeared to have unusual binding behavior, potentially due to binding of two monomers of IL-17. Error bars represent the standard deviation for N=3 trials.

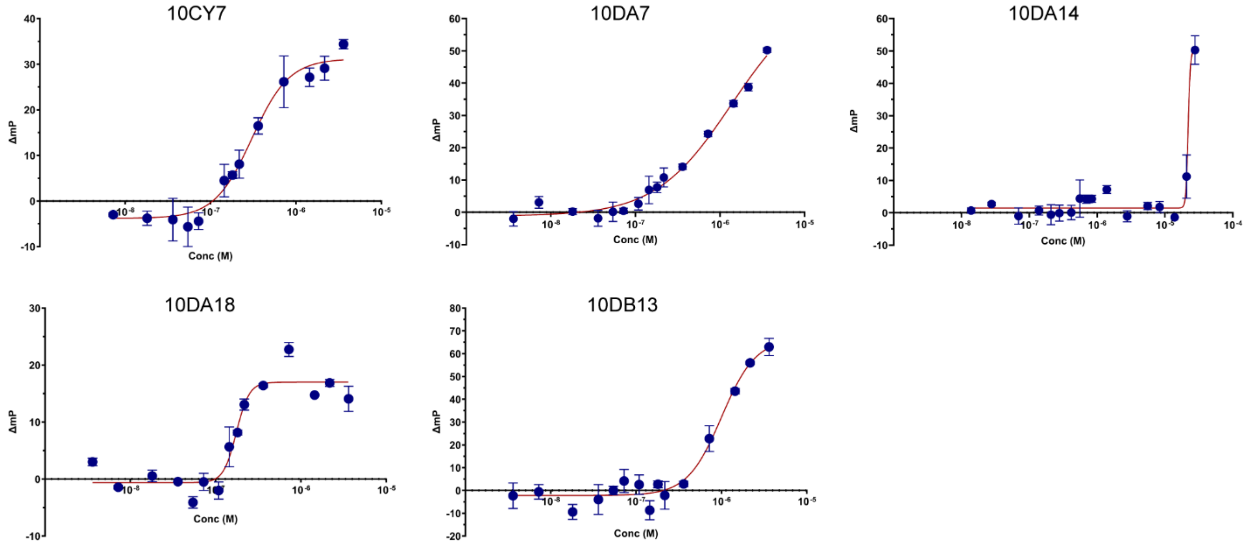

Figure S4. Fluorescence polarization of IL-20 binding aptamers. Binding constants were determined from non-linear fits using GraphPad Prism as described. Error bars represent the standard deviation for N=3 trials.

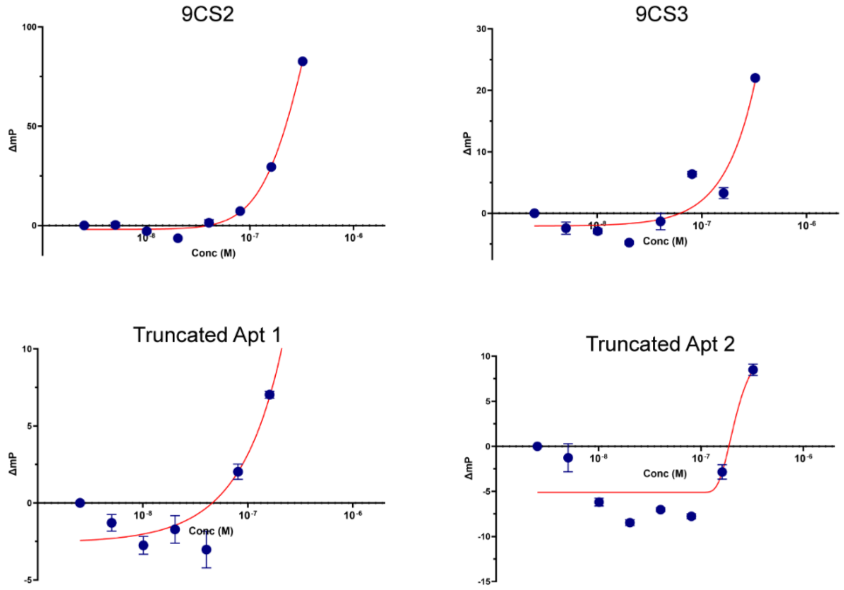

Figure S5. Fluorescence polarization of IL-17 binding aptamers with mouse IL-17 Binding curves were plotted with non-linear fits using GraphPad Prism as described. Error bars represent the standard deviation for N=3 trials.

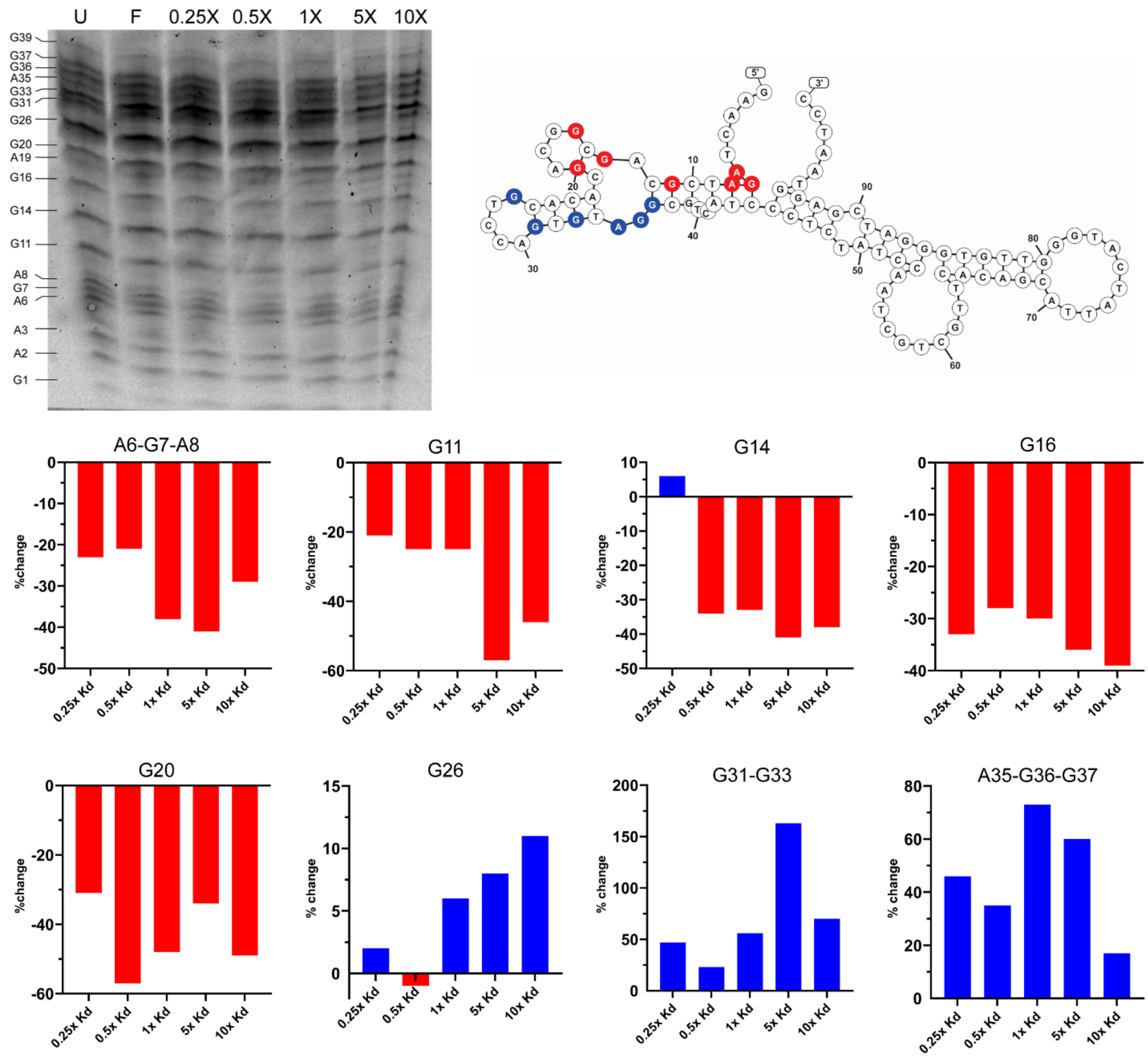

Figure S6. DMS probing of aptamer 10CZ1. 5' TAMRA-labeled 10CZ1 was incubated with increasing concentrations of IL-20 and DMS cleavage patterns were compared to unfolded (U) and folded (F) aptamer without IL-20. K_d for aptamer 10CZ1 was determined to be 120 nM via fluorescence polarization. The percent change refers to the change in band intensity relative to the folded aptamer lane. Increased cleavage, resulting in a stronger band intensity, is shown in blue, while decreased cleavage is represented in red. In some cases, groups of bands were quantified as a single unit. Gel image was analyzed using Image Lab. Secondary structure prediction was generated with SnapGene® software (from Dotmatics; available at snapgene.com).

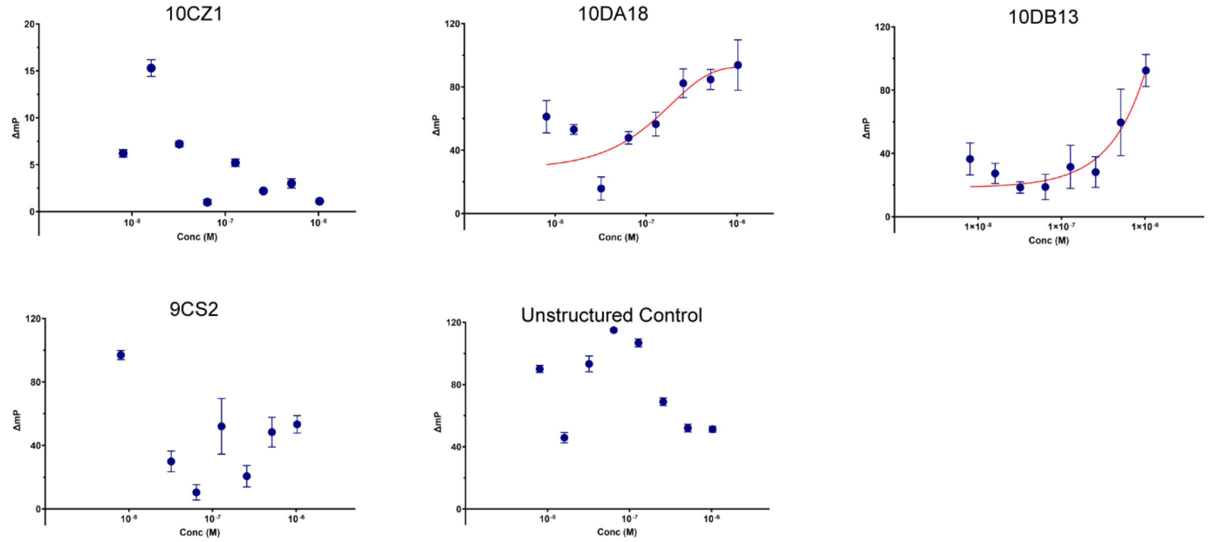

Figure S7. Fluorescence polarization of aptamers with IL-24 Binding curves were plotted with non-linear fits using GraphPad Prism as described. Error bars represent the standard deviation for N=3 trials.
